# Juvenile idiopathic arthritis (JIA) in two tertiary centres in the Western Cape, South Africa

**DOI:** 10.1186/1546-0096-9-S1-P136

**Published:** 2011-09-14

**Authors:** Kate Weakley, Monika Esser, Chris Ranier Pope, Christiaan Scott

**Affiliations:** 1 Red Cross Childrens Hospital, University of Cape Town, Cape Town, South Africa; 2 Tygerberg Hospital, Stellenbosch University, Cape Town, South Africa; 3 Retired, Cape Town, South Africa

## Background

JIA is a disease that shows wide variations between differing populations. Due to recent international consensus on classification criteria, JIA has been widely described in many countries and population groups. There has been almost no data that describes JIA in an African, specifically Sub-Saharan African setting.

## Objective

To describe disease characteristics and functional disability in two tertiary centres in the Western Cape, South Africa

## Methods

87 children were recruited during random clinic visits to rheumatology clinics at Tygerberg and Groote Schuur Hospital between April 2010 and April 2011. Children were diagnosed using International League of Associations for Rheumatology (ILAR) 2001 classification criteria. Consent was obtained, medical records examined, Childhood Health Assessment Questionnaires (CHAQ), visual analogue scales (VAS) for pain and general wellbeing were completed and all children were examined by a researcher in conjunction with a paediatric rheumatologist. HIV status as well as tuberculosis disease and treatment were investigated.

## Results

A total of 87 children were enrolled. 9 children were excluded (2 HIV arthropathy, 1 TB arthritis, 1 SLE, 4 insufficient data, 1 fibromyalgia) leaving a total of 78 patients. There was an equal female to male ratio-39 males and 39 females. There were 6 Systemic JIA (7.69%), 17 Oligoarthritis (21,79%), 11 Polyarthritis rheumatoid factor (RF) positive (14.10%), 21 Polyarthritis RF negative (26.9%), 1 psoriatic arthritis (1.28%), 18 enthesitis related arthritis (23%) and 4 Oligoextended JIA (5.12%). The mean CHAQ for the group was 0.5, the mean VAS for pain was 1.8cm and mean VAS for general wellbeing was 2.5cm. Kruskal-Wallis equality-of-populations rank test showed significant differences (p<0.05) between CRP, ESR, number of active joints and limited joints between subtypes.

## Conclusion

JIA has a unique epidemiology in the Western Cape of South Africa, with equal male:female predominance and increased rates of Polyarticular RF positive and Enthesitis related Arthritis. Factors such as TB and HIV complicate the issue of JIA in a Sub-Saharan African setting.

